# VHH-Photosensitizer Conjugates for Targeted Photodynamic Therapy of Met-Overexpressing Tumor Cells

**DOI:** 10.3390/antib8020026

**Published:** 2019-04-04

**Authors:** Raimond Heukers, Vida Mashayekhi, Mercedes Ramirez-Escudero, Hans de Haard, Theo C. Verrips, Paul. M.P. van Bergen en Henegouwen, Sabrina Oliveira

**Affiliations:** 1 QVQ Holding BV, Yalelaan 1, 3584 CL Utrecht, The Netherlands; r.heukers@qvquality.com (R.H.); theo.verrips@outlook.com (T.C.V.); 2 Cell Biology Division, Department of Biology, Faculty of Science, Utrecht University, Padualaan 8, 3584 CH Utrecht, The Netherlands; v.mashayekhi@uu.nl (V.M.); s.oliveira@uu.nl (S.O.); 3 Crystal & Structural Chemistry, Bijvoet Center for Biomolecular Research, Faculty of Science, Utrecht University, Padualaan 8, 3584 CH Utrecht, The Netherlands; m.ramirezescudero@uu.nl; 4 Argenx BVBA, Industriepark-Zwijnaarde 7, 9052 Gent, Belgium; hdehaard@argenx.com; 5 Pharmaceutics Division, Department of Pharmaceutical Sciences, Faculty of Science, Utrecht University, Universiteitsweg 99, 3584 CG Utrecht, The Netherlands

**Keywords:** targeted photodynamic therapy, hepatocyte growth factor receptor, HGFR, c-Met, Met, nanobodies, VHH, photosensitizer

## Abstract

Photodynamic therapy (PDT) is an approach that kills (cancer) cells by the local production of toxic reactive oxygen species upon the local illumination of a photosensitizer (PS). The specificity of PDT has been further enhanced by the development of a new water-soluble PS and by the specific delivery of PS via conjugation to tumor-targeting antibodies. To improve tissue penetration and shorten photosensitivity, we have recently introduced nanobodies, also known as VHH (variable domains from the heavy chain of llama heavy chain antibodies), for targeted PDT of cancer cells overexpressing the epidermal growth factor receptor (EGFR). Overexpression and activation of another cancer-related receptor, the hepatocyte growth factor receptor (HGFR, c-Met or Met) is also involved in the progression and metastasis of a large variety of malignancies. In this study we evaluate whether anti-Met VHHs conjugated to PS can also serve as a biopharmaceutical for targeted PDT. VHHs targeting the SEMA (semaphorin-like) subdomain of Met were provided with a C-terminal tag that allowed both straightforward purification from yeast supernatant and directional conjugation to the PS IRDye700DX using maleimide chemistry. The generated anti-Met VHH-PS showed nanomolar binding affinity and, upon illumination, specifically killed MKN45 cells with nanomolar potency. This study shows that Met can also serve as a membrane target for targeted PDT.

## 1. Introduction

Photodynamic therapy (PDT) is a type of cancer treatment in which tumor cells are killed by reactive oxygen species, such as singlet oxygen, formed by the local and light-induced activation of a photosensitizer (PS) [1]. By locally reacting with proteins, lipids, and nucleic acids, the reactive oxygen species generated can induce cell death, vascular damage, and an inflammatory response [2]. It is for this mode of action that PDT is used in clinic to treat malignancies. Unfortunately, the PSs generally used in the clinic are relatively hydrophobic, are systemically applied, and are non-targeted. These factors combined can result in off-target toxicity and long lasting photosensitivity [2,3]. Improved strategies involve the development of more water-soluble PS, delivery of PS with nanocarriers and photo-immunotherapy (PIT), among others [4,5,6]. PIT is one of the strategies of targeted PDT, in which the PS is delivered selectively to tumors via conjugation to tumor-targeting antibodies.

In order to increase the efficacy of targeted PDT and reduce the period of photosensitivity, we have recently introduced smaller PS conjugates with enhanced tumor penetration in combination with reduced blood circulation time [7,8]. These smaller conjugates were generated by conjugating a water-soluble PS to small antibody fragments originating from heavy chain antibodies found in animals of the *Camelidae* family (i.e., nanobodies, single domain antibodies (sdAbs) or VHHs (variable domain of the heavy chain from heavy chain-only antibodies)) [9]. Compared to commonly used conventional antibodies of the IgG class, VHHs are 10 times smaller and consist of only a single domain with in general only one, rarely two, disulfide bridges [10,11]. These features favor the selection and production process and make VHHs very stable [11]. Also, because the C-terminus of a VHH is located opposite to its epitope-binding loops (i.e., complementary determining regions), C-terminal conjugation to effector molecules generally does not affect the binding properties [12,13,14,15,16].

Previously, we have described the nanobody-PS or VHH-PS conjugates specifically targeting the epidermal growth factor receptor (EGFR), a receptor tyrosine kinase (RTK) that is found overexpressed on a large variety of cancers, such as head and neck, lung, or colon cancer [17,18]. In vitro, upon illumination, VHH-PS conjugates selectively killed EGFR-overexpressing tumor cells with nanomolar IC_50_ values. In vivo, VHH-targeted PDT induced 80%–90% tumor necrosis, as measured 24 h after illumination [7,8]. Another receptor tyrosine kinase, which is frequently overexpressed or deregulated in a large number of carcinomas, sarcomas, hematopoietic malignancies, and other neoplasms is the hepatocyte growth factor receptor (HGFR, c-Met or Met) [19,20]. Met is a 195 kDa hetero-dimeric single-membrane spanning receptor tyrosine kinase that is activated by hepatocyte growth factor (HGF, also known as scatter factor) [21]. The extracellular part of Met consists of three subdomains: an N-terminal 7-bladed β-propeller-like SEMA (semaphorin-like) subdomain, a PSI (plexin, semaphorin, integrin-like) subdomain, and four IPT (immune-globulin-like, plexins, transcription factors) subdomains [22,23]. Blades 2–3 of the SEMA subdomain and IPT 3–4 interact with its natural ligand HGF [24]. In a previous study, multiple VHHs targeting the extracellular part of Met were developed (i.e., E9, F5, and G2) [25]. Of these VHHs, G2 was used to target cross-linked albumin nanoparticles to Met-expressing cells [25]. By using the anti-Met VHH G2, the targeted nanoparticles showed specific binding and uptake into Met-expressing cells.

In this study, we have characterized these Met-targeting VHHs further by assessing the subdomains they bind to. Moreover, we have used the best of these VHHs for the specific delivery of water-soluble PS to Met-expressing cells for targeted PDT. That VHH was extended with a C-terminal C-Direct tag, allowing affinity purification from yeast supernatant and directional conjugation of the PS to an unpaired cysteine using maleimide chemistry. Subsequently, binding of this conjugate to cells and their ability to kill Met-expressing cells by PDT were evaluated.

## 2. Methods

### 2.1. Molecular Modeling

The molecular structure of G2c with the C-Direct tag was modeled with I-TASSER [26] and visualized using the PyMOL Molecular Graphics System, Version 2.0 (Schrödinger, LLC, Cambridge, USA). Nb10 from PDB ID 4DKA:A showed 85% homology to G2 and was taken as a reference [27].

### 2.2. VHH Production and Purification

Selection and characterization of VHH clones targeting Met have been described previously [25]. VHH protein for epitope mapping was obtained from production in *E. coli* TG1. For this, the VHH genes were cloned into the pMEK222 vector for productions in *E. coli*, which provides the VHH with a C-terminal FLAG-His tag. VHHs were produced and purified from *E. coli* TG1 using immobilized metal-affinity chromatography (IMAC, Thermo Fisher Scientific, Waltham, MA, USA) as previously described [7,28]. For production in yeast, VHH genes were recloned in the pYQVQ11 vector for VHH production in yeast, which provides the VHH with a C-terminal C-Direct tag containing a free thiol (cysteine) and an EPEA (Glu, Pro, Glu, Ala) purification tag (C-tag, Thermo Fisher Scientific). To improve production yields and facilitate purification from supernatant, C-Direct-tagged VHH were produced in *S. cerevisiae* strain VWK18 as described previously [28,29,30,31]. VHHs were purified from the yeast supernatant using an Äkta Start (GE Healthcare, Chicago, IL, USA), a Capture-Select affinity chromatography column (Thermo Fisher Scientific) and size-exclusion chromatography (Thermo Fisher Scientific) according to the manufacturer’s protocols. Purified VHH was filter sterilized and stored in PBS (phosphate buffered saline).

### 2.3. Cell Culture

Met-overexpressing human gastric cancer cell line MKN45 (ACC-409) was obtained from the Deutsche Sammlung von Mikroorganismen und Zellkulturen (DSMZ, Braunschweig, Germany). The human ovarian carcinoma cell line TOV112D (CRL-11731) was obtained from the American Tissue Culture Collection (ATCC, LGC Standards GmbH, Wesel, Germany). TOV112D cells that stably express Met (TOV + Met) were previously described [25]. These cell lines were cultured as previously described in either DMEM (Dulbecco’s Modified Eagle Medium) (Thermo Fisher Scientific) for TOV112D cells or RPMI 1640 (Thermo Fisher Scientific) for MKN45 cells [25]. HepG2 cells were obtained from ATCC (LGC Standards GmbH) and were cultured in DMEM low-glucose (Thermo Fisher Scientific). All media was supplemented with streptomycin, penicillin, l-glutamine, and FCS (fetal calf serum) as described previously [25].

### 2.4. VHH Binding ELISA

Binding affinity and binding epitopes were determined by ELISA on the Met ectodomain. The human Met, llama Met, llama/human chimeric Met ectodomains, and control antibody c224G11 were described previously [32]. Maxisorp 96-wells plates (Thermo Fisher Scientific) were coated overnight with 100 μL of 2 μg/mL of ectodomain and subsequently blocked with 4% milk in PBS. Bound c224G11 was detected with donkey-anti-mouse^HRP^ (Bio-Rad, Hercules, CA, USA), and bound VHH was detected using mouse-anti-FLAG (clone M2, Sigma-Aldrich, St. Louis, MO, USA) followed by donkey-anti-rabbit^HRP^ (Bio-Rad) with TMB Ultra as substrate (Thermo Fisher Scientific). Antibody incubations were performed in 1% milk in PBS and for 1 h at room temperature. Absorbance was measured using a Multiskan Go plate reader (Thermo Fisher Scientific). Binding of PS-conjugated VHH was carried out in cells in binding medium (DMEM without phenol red, 50 mM HEPES pH 7.5, supplemented with 1% bovine serum albumin) for 2 h at 4 °C to prevent internalization. Bound VHH-PS was detected using the Odyssey near-infrared scanner (LI-COR Biosciences, Lincoln, NE, USA). Data was plotted and fitted for one-site-specific binding using Prism 7 software (GraphPad, La Jolla, CA, USA).

### 2.5. Surface Plasmon Resonance (SPR)

Met ectodomain variants (human, llama and chimera LS1 and LS5) were amine-coupled on a pre-activated G-COOH sensor chip (SensEye) with a Continuous Flow Microspotter (CFM, Wasatch Microfluidics Inc., Salt Lake City, UT, USA) at pH 4.5 (50 mM Sodium acetate buffer, 0.005% Tween 20). Beforehand, the sensor chip was equilibrated in 50 mM MES (4-Morpholineethanesulfonic acid) buffer pH 5.5, then activated with 400/100 mM EDC/NHS [1-ethyl-3-(3-dimethylaminopropyl) carbodiimide hydrochloride/N-Hydroxysuccinimide]. After ectodomain coupling, excess reactive esters were deactivated with 100 mM ethanolamine pH 8. The amount of immobilized protein ranged from 700 to 3000 response units (RU); 1 RU corresponds to approximately 1 pg of protein per mm^2^. Surface plasmon resonance (SPR) experiments were performed at a constant temperature of 25 °C on a MX96 (IBIS Technologies). G2 was flowed over the sensor chip as analyte at concentrations ranging from 20 pM to 200 nM for 60 min in buffer containing 25 mM phosphate-buffered saline pH 8.0 and 0.005% Tween 20. After each injection, an 8 min dissociation time was set, followed by a regeneration step of 30 s with 50 mM acetic acid buffer, pH 4.5. Calibration of sensor signals and reference subtraction was evaluated with SprintX (IBIS Technologies, Enschede, The Netherlands), and further analyses were performed in Scrubber 2 (BioLogic Software, Seyssinet-Pariset, France). Association- and dissociation-rate constants (k_a_/k_d_) were determined by globally fitting the SPR data to a 1:1 Langmuir binding model. The dissociation constant (K_D_) was calculated from the k_a_ and k_d_ parameters. In addition, the K_D_ was determined from the steady state analyte binding levels (averaged between 40 and 60 min association time) plotted against concentration, and by fitting a one-site saturation model.

### 2.6. Conjugation to PS

VHHs were site-directionally conjugated to the photosensitizer IRDye700DX–maleimide (LI-COR Biosciences). First, the VHHs were incubated with an 2.75-fold molar excess of TCEP (tris(2-carboxyethyl) phosphine hydrochloride) (VWR International, Radnor, PA, USA) to reduce the C-terminal cysteine upon which the VHH were incubated with a four-fold molar excess of IRDye700DX-maleimide for 16 h at 4 °C. Free label was removed by size-exclusion chromatography using three consequent Zeba Desalting Columns (Thermo Fisher Scientific) according to the manufacturer’s protocols. Degree of conjugation was determined using the Multiskan Go spectrophotometer (Thermo Fisher Scientific), and the amount of free dye was determined after size separation by SDS-PAGE (Bio-Rad) on the Odyssey scanner (Li-COR Biosciences). Afterwards, the SDS-PAGE gel was stained with Page Blue (Thermo Fisher Scientific) to show total protein. For the internalization assay, G2c was conjugated to HiLyte Fluor 647 (HL647)–maleimide (Eurogentec, Liege, Belgium) according to the protocol described above.

### 2.7. Internalization Assay

MKN45 cells were seeded in eight-well chamber slides (Lab-Tek, Nunc, Thermo Fisher Scientific) two days before the experiment. Cells were incubated with 1 µM G2-Alexa647 conjugate for 2 h at 37 °C. Unbound conjugate was removed by washing three times with PBS. Cells were fixed in 4% PFA for 10 min at room temperate (RT). PFA-induced autofluorescence was quenched using 100 mM glycine in PBS (10 min, RT). Cells were washed with PBS and then incubated with DAPI (4′,6-diamidino-2-phenylindole, 0.25 µg/mL, Thermo Scientific) for 10 min at RT. The slides were mounted using SlowFade (Invitrogen) and pictures were taken with a LSM700 confocal microscope using a 63× oil immersion objective (Carl Zeiss Microscopy, Jena, Germany). The images were analyzed with ImageJ.

### 2.8. Photodynamic Therapy

Photodynamic therapy was performed similarly to the process previously described [7,8]. Cells (24,000/well) were seeded in 96-well cell culture plates (Greiner) and allowed to adhere overnight. Cells were then pulsed with VHH-PS in medium without phenol red for 2 h at 37 °C, after which unbound VHH-PS was removed by washing the cells twice with 100 μL medium. Bound VHH-PS was then detected using the Odyssey scanner and an EVOS fluorescence microscope (Thermo Fischer Scientific). Cells were illuminated for 1 h using a 690 diode laser (Modulight, Tampere, Finland) with a 5 mW/cm^2^ fluence rate for a total light dose of 18 J/cm^2^, then were incubated overnight at 37 °C in the cell culture incubator. The next day, the cells were screened for viability. For this, cells were incubated with calcein (Thermo Fisher Scientific) and propidium iodide (Thermo Fisher Scientific) to stain live and dead cells, after which they were imaged on an EVOS fluorescence microscope. Cell viability was quantified using the alamarBlue reagent (Thermo Fisher Scientific), which was quantified using a FLUOstar Optima plate reader (BMG Labtech, Ortenberg, Germany). Data were plotted and fitted using Prism 7 software (GraphPad, La Jolla, CA, USA).

## 3. Results

**Figure 1:**
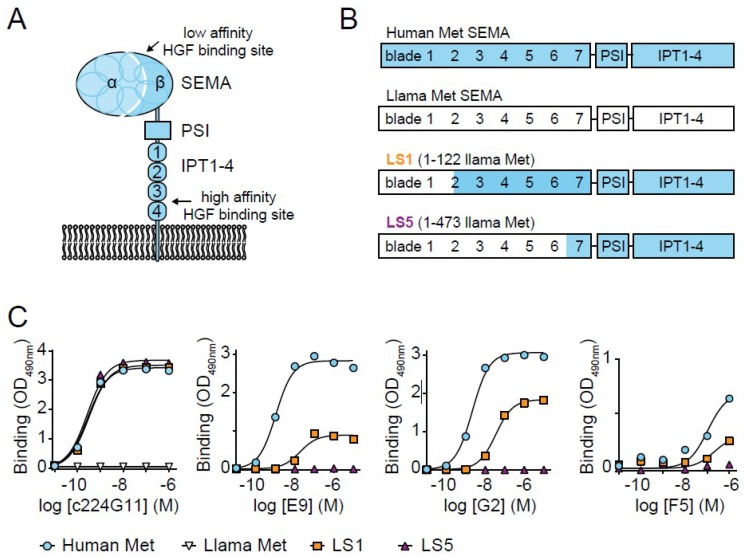
Selected VHHs (variable domains from the heavy chain of llama heavy chain antibodies) recognize SEMA (semaphorin-like) subdomain on human Met ectodomain. (**A**) Met ectodomain consisting of a large SEMA subdomain (a cleaved seven-bladed propeller alpha and beta subunit), a PSI (plexin, semaphorin, integrin-like) subdomain, and four IPT (immune-globulin-like, plexins, transcription factors) subdomains. HGF (hepatocyte growth factor) binds with low affinity to the SEMA subdomain and with high affinity to the IPT subdomains. (**B**) Schematic representation of human (blue) and llama (white) Met and the two chimeric Met variants LS1 and LS5 (llama SEMA) in which either the first 122 or 473 amino acids of the human Met were replaced with those from llama. (**C**) Representative figure of binding of either the conventional control antibody c224G11 or the VHHs E9, G2, or F5 to the four Met variants in ELISA (*n* = 2).

Three previously selected VHHs recognizing the Met ectodomain were taken for further characterization, i.e., E9, G2 and F5 [25]. These VHHs were recloned in an *Escherichia coli* production vector, and binding to the human Met ectodomain was assessed with ELISA (Figure 1A–C). The two VHH clones E9 and G2 bound to the human Met ectodomain with apparent binding affinities (K_D_) of 7 ± 8 nM and 4 ± 3 nM, respectively. The K_D_ value of F5 could not be determined properly, but was estimated to be at least higher than 50 nM. To investigate which subdomain these VHHs bind to, ELISAs on chimeric human/llama Met ectodomains were performed. In these chimeric constructs (LS, llama SEMA), either the first 122 (LS1) or 473 (LS5) amino acids of human Met were replaced by those of llama Met (Figure 1B) [32]. Integrity of these constructs was confirmed by the binding of the anti-Met antibody c224G11 (ABT-700 or Telisotuzumab), which is directed against the first IPT region (Figure 1C, left) [32,33,34]. c224G11 showed a similar binding affinity to both human Met and the two chimeric mutants, whereas no binding of c224G11 to llama Met was observed. Because the VHHs were raised in llama, no cross-reactivity of the VHHs with llama Met or parts of llama Met was expected. The binding affinity and B_max_ of all three VHHs was completely lost for LS5 and strongly reduced on LS1, as compared to human Met, suggesting that the binding epitopes for all three VHHs involve propeller blades 2–6 of the SEMA subdomain. Due to its high affinity for Met and the additional characterization performed previously [25], G2 was selected as the lead clone for Met-targeted PDT.

**Figure 2:**
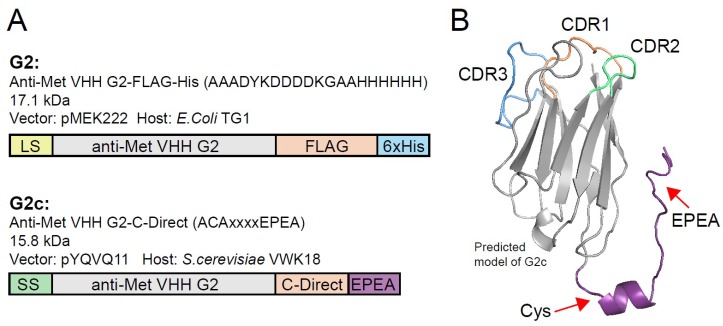
Production of G2 with a C-terminal tag containing a free thiol (G2c) and conjugation to PS. (**A**) Schematic representation of the anti-Met VHH G2, expressed as a FLAG-His tagged protein (top) and G2c carrying an additional cysteine in its C-terminal C-Direct tag. The molecular weights of the proteins are indicated, as well as the expression vector and production organism used. LS, pelB leader sequence. SS, Suc2 secretion sequence. (**B**) Predicted model of G2c in which the C-terminal tag (purple) containing the unpaired cysteine and the EPEA (Glu, Pro, Glu, Ala) affinity tag is indicated with red arrows. CDRs (complementarity determining regions) are located on the opposing end of the VHH. The model was based on the structure of Nb10 (85% amino acid sequence homology with QME-G2) as published by Park et al. (PDB ID 4DKA:A, C-score 0.13) [27].

In order to facilitate directional conjugation of photosensitizers to VHHs without affecting their binding integrity, an additional cysteine was introduced to the C-terminus of G2 via incorporation into the C-terminal C-Direct tag, thereby creating G2c (Figure 2A). Molecular modeling of G2c was performed based on the published structure of a VHH with high homology (Nb10, PDB ID 4DKA:A, C-score 0.13) [27]. This model showed that the unpaired cysteine in the C-Direct tag is located opposite of the CDRs (complementarity determining regions) and close to its framework, which should allow functionalization of VHHs without affecting their binding characteristics (Figure 2B). For higher yield productions, G2c was produced in *Saccharomyces cerevisiae* and purified from supernatant using affinity chromatography and size-exclusion chromatography.

**Figure 3:**
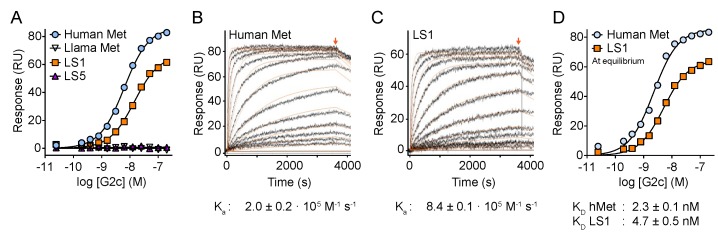
Binding analysis of G2c on Met using surface plasmon resonance (SPR). (**A**) Binding of G2c to wild-type human Met ectodomain and the LS1 llama/human chimera, but not the llama or the LS5 chimeric Met ectodomain proteins. (**B**,**C**) Representative SPR sensorgrams of the association phase and dissociation phase (8 min) (starting at arrow) of G2c binding to human Met (**B**) and LS1 chimera (**C**). Kinetic fitting is shown in orange. (**D**) Equilibrium-binding plot of G2c to human Met and LS1 chimeric variant. RU refers to response units. Kinetic parameters and equilibrium dissociation constants are the average ± SD of three independent measurements.

We examined the propensity of G2c to interact with the four variants of the Met ectodomain (human, llama, LS1 and LS5) using surface plasmon resonance (SPR). In agreement with the ELISA data (Figure 1), the SPR experiments confirm that G2c interacts with both human Met and the LS1 variant but not with llama Met or the LS5 variant (Figure 3A), suggesting the involvement of propeller blades 2–6 of the Met SEMA subdomain to be involved in the binding of G2c. The association constant was four times lower towards LS1 compared with human Met (Figure 3B,C). Kinetic parameters calculated at equilibrium conditions (Figure 3D) showed that the binding affinity of G2c for LS1 is lower as compared with its affinity to human Met (K_D_ of 2.3 ± 0.1 nM for human Met and 4.7 ± 0.5 nM for LS1, *n* = 3). In conclusion, addition of a cysteine residue to the C-terminal region of G2 did not affect binding affinity to human Met nor did it affect domain specificity.

**Figure 4:**
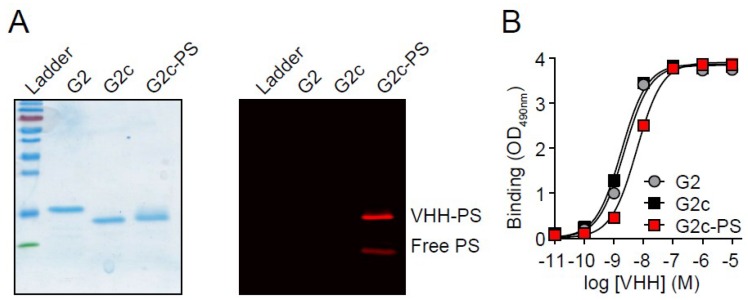
Conjugation of G2c to photosensitizer IRDye700DX. (**A left**) Reducing SDS-PAGE gel showing purified G2 from *Escherichia coli*, G2c from *Saccharomyces cerevisiae*, and G2c-PS. Size-separated proteins were stained with PageBlue stain. (**A right**) G2c-PS and free PS in the gel shown on the left, as detected with an Odyssey scanner before the PageBlue stain. (**B**) Binding of G2, G2c, and G2c-PS to Met ectodomain in ELISA (representative figure, mean ± SD).

**Figure 5:**
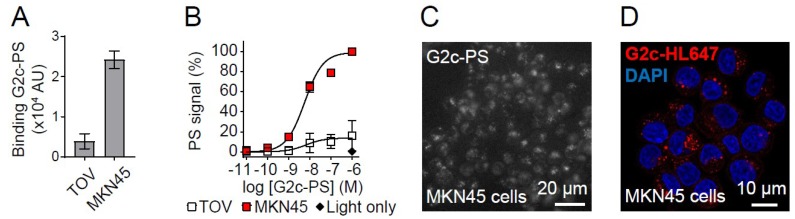
Met-targeting G2c-PS conjugates associate with Met-overexpressing MKN45 cells. (**A**) Binding of G2c-PS to MKN45 cells and not to TOV112D cells. Cells were incubated with 50 nM G2c-PS for 2 h on ice and PS fluorescence was detected with an Odyssey infrared scanner. Mean ± SD of *n* = 2 is shown. (**B**) Increased cell association of G2c-PS with MKN45 cells with increasing concentrations. MKN45 and TOV112D cells were pulsed with G2c-PS for 2 h at 37 °C. PS fluorescence was detected with an Odyssey infrared scanner. Mean ± SD of *n* = 3 is shown. (**C**) Association of G2c-PS in MKN45 cells. Cells were pulsed for 2 h with 1 μM of G2c-PS and imaged by wide-field fluorescent microscopy. (**D**) Uptake of G2c-HL647 in MKN45 cells. Cells were pulsed for 2 h with 1 μM of G2c-HL647 (red) and imaged by confocal laser scanning microscopy.

**Figure 6:**
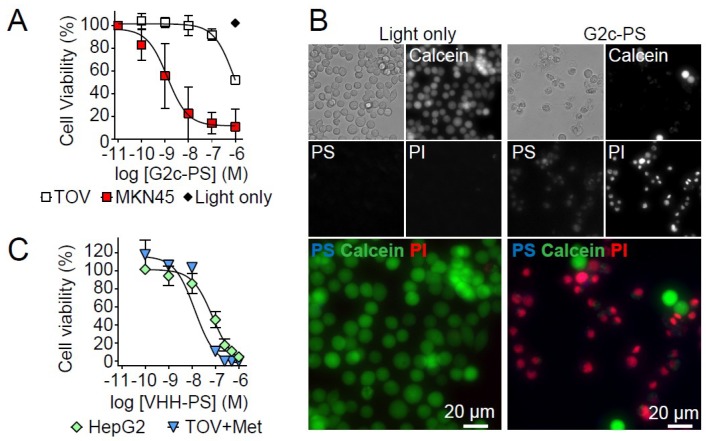
Met-targeting G2c-PS induce cell death of Met-expressing cells upon activation by illumination. (**A**) Selective killing of Met-overexpressing MKN45 tumor cells and not of Met-negative TOV112D cells by G2c-targeted PDT. Cells were pulsed for 2 h at 37 °C and subsequently illuminated to activate the PS. Cell viability was assessed one day later. Mean ± SD of *n* = 3 is shown. (**B**) Phase contrast and fluorescence microscopy (EVXOS) of PDT-treated MKN45 cells showing G2c-PS (blue in merge, visible as magenta (red + blue)), live cells (calcein, green), and dead cells (propidium iodide, red). (**C**) Induction of cell death of Met-expressing TOV + Met and HepG2 cells by G2c-PS upon a 2 h pulse followed by PS activation via illumination. Representative plot of *n* = 2.

Subsequently, the free thiol in G2c was conjugated to the water-soluble PS IRDye700DX using maleimide chemistry, resulting in a degree of conjugation of ~1, as determined spectrophotometrically. However, because ~40% of the signal in the solution was still free PS (Figure 4A), a degree of conjugation of ~0.6 would be a more realistic estimation. Because of the hydrophilic nature of IRDye700DX and the lack of toxicity observed for free PS in our previous study [7], we decided to continue with the conjugated G2c-PS. Conjugation of PS to G2c only mildly affected its apparent binding affinity for Met, as determined by ELISA (with apparent affinity values of 2.2 ± 0.2 nM for G2, 1.8 ± 0.1 nM for G2c, and 5.9 ± 0.3 nM for G2c-PS, Figure 4B). In addition, G2c-PS was also still able to bind to the Met-overexpressing MKN45 cells (Figure 5A). No binding of G2c was detected on Met-negative TOV112D cells. For the in vitro PDT experiments, cells were pulsed with a concentration range of G2c-PS for 2 h at 37 °C, a pulse duration that reflects the circulation of VHHs in blood and the time required for tumor uptake of fluorescently labeled VHHs [35]. Besides acting as a PS via the production of reactive oxygen species, IRDye700DX is also a fluorophore and can therefore be used for detecting binding of the conjugate to cells. During the pulse, G2c-PS was able to associate with Met-expressing MKN45 cells with half-max signals being obtained at concentrations comparable to the binding affinity of the VHH for the Met ectodomain (Figure 5B). Little association of G2c-PS was observed for the Met-negative TOV112D cells. Uptake of G2c-PS by MKN45 cells was initially suggested in wide-field microscopy images (Figure 5C), which was confirmed by confocal microscopy imaging (Figure 5D). Exposure of MKN45 cells to different concentrations of G2c-PS, combined with illumination, resulted in ~0% cell viability at nanomolar concentrations (Figure 6A). This was also confirmed by fluorescence microscopy, in which almost all cells that were pulsed with G2c-PS and subsequently illuminated showed an uptake of propidium iodide and an absence of calcein staining (Figure 6B). We subsequently assessed the ability of G2c-PS to induce cell death of two other Met-expressing cell lines. Although showing lower EC50 values compared with MKN45 cells, G2c-PS could reduce the viability of the previously described TOV + Met cell line and the liver hepatocellular carcinoma cell line HepG2 in a dose-dependent manner (Figure 6C).

## 4. Discussion

PDT is a valuable method for inducing local cell death of malignant cells by the local activation of PS. Unfortunately, clinically employed PS are administered systemically, are relatively hydrophobic, and passively accumulate in the tumor as a result of their hydrophobicity in combination with the enhanced permeability and retention (EPR) effect [36,37]. This results in a sub-optimal tumor uptake of PS, off-target toxicity, and long photosensitivity [38]. To enhance the specific tumor uptake of photosensitizers, PS-delivering nanocarriers have been developed and water-soluble PSs have been conjugated to tumor-specific peptides and antibodies (the latter is also known as photo-immunotherapy, or PIT) [39,40,41]. Because the extent of damage caused by PDT is correlated to the amount of PS delivered, ideal targets for these approaches are highly expressed on tumor cells and preferably in a homogeneous fashion throughout the solid tumor mass. Examples of tumor targets are the typical tumor-related receptors EGFR [42,43] and HER2 [44], although the interleukin-2 receptor [43] and carcinoembryonic antigen have also been used as valuable tumor biomarkers [45,46,47]. In some types of cancers (e.g., non-small cell lung cancer, or colorectal cancer), treatment with anti-EGFR therapy has resulted in the intratumoral clonal selection of therapy-resistant cells [48]. This can be the result of activating mutations of signaling proteins from the EGFR signaling pathways, such as KRAS. Alternatively, other receptor tyrosine kinases (RTK) can become activated and, in most of these cases, upregulation of the Met receptor tyrosine kinase is regarded as one of the main mechanisms for this type of resistance [49,50,51,52]. (The Met RTK is also considered a good tumor target, and therapeutic anti-Met antibodies have been developed [32,34,53].) In order to expand possible therapeutic strategies for certain cancers, we here evaluated whether Met could also serve as a target for targeted PDT.

Targeted PDT using antibodies as targeting agents (i.e., PIT) is currently being clinically evaluated for the treatment of recurrent head and neck cancer (ClinicalTrials.gov: NCT02422979). These results are eagerly awaited, as these are the first tests in patients and thus could significantly advance the field of targeted PDT. Nevertheless, we consider further improvements a necessity, and in the current study we have employed nanobodies or VHHs as targeting agents. Conventional antibodies of the IgG-type are large, dimeric molecules, typically designed for extended blood circulation. These characteristics may lead to slow tumor penetration/accumulation, poor tumor to normal tissue ratios, and long photosensitivity [54]. Reduction of the duration of photosensitivity might be achieved by the rapid tumor accumulation and subsequent rapid clearance of PS from the body. Antibody fragments smaller than IgG like Fabs, scFvs, or VHH/nanobodies have these properties without compromising their affinity for their tumor biomarkers [55]. As we have described before, VHHs in particular are characterized by rapid tumor accumulation and rapid blood clearance, combined with the ability to bind targets with high affinity [16,35]. Another argument for using VHHs over conventional antibody fragments might be the short distance between the conjugated PS and the paratope on the target (~4 nm). Because the reactive oxygen species generated by PS are short lived (<40 ns) and may travel only a short distance (<20 nm), the delivery of the PS close to the cell membrane or to vital cell organelles might favor its potency [56,57]. For that same reason, it would be of interest to assess the potency of Met-targeting PS conjugates recognizing epitopes closer to the membrane than the SEMA subdomain, such as the PSI or IPT subdomains [23]. This would require new PSI- or IPT-specific VHHs.

By using chimeric constructs consisting of different human and Llama Met fragments, we were able to show that all three Met-targeting VHHs used in this study bind to the SEMA subdomain of the Met ectodomain. The best SEMA binder (i.e., G2) was provided with a C-terminal tag containing an unpaired cysteine to allow conjugation to the PS using maleimide chemistry. The addition of this thiol-containing C-Direct tag did not affect the binding characteristics of the VHH, which is in line with previous studies of C-terminal-labeled VHHs via an unpaired cysteine [14,15,16]. Multiple methods have been described to site-directional functionalize antibody fragments [15,58]. In our experience, incorporation of an unpaired cysteine in the C-terminal tag allowed the straightforward production, purification, and conjugation to commercially available tracers with the use of a single tag [15,16,59].

The binding affinity of G2c for Met was determined on purified ectodomains using two different technologies: ELISA and SPR. In these experiments, the apparent binding affinity of G2c in ELISA was comparable (low nanomolar range) to the K_D_ values determined under equilibrium by SPR. The SPR analysis does, however, allow the determination of association and dissociation rate constants, which provided additional information on the binding kinetics of G2c on wild-type and chimeric Met proteins. Moreover, similar affinities for G2 were found for binding to Met-expressing cells [25], indicating that cellular components do not influence VHH binding.

Application of the anti-Met conjugate G2c-PS in in vitro PDT resulted in the efficient and specific killing of the Met-overexpressing MKN45 cells and the Met-expressing TOV + Met and HepG2 cells, while the Met negative cell line TOV112D remained undisturbed. The observation that the treated cells could be stained with propidium iodide suggests that cells could have died through necrosis. Further studies would be needed to determine the exact mechanism of cell death. The potency of G2c-PS in killing MKN45 cells was in the nanomolar range. In the two other cell lines, the Met-expressing HepG2 tumor cells and the TOV112D cells stably expressing Met (TOV + Met), the observed potencies were lower. These differences in potencies might be related to the relative Met expression levels of these cells. MKN45 is a highly Met-overexpressing cell line due to genomic amplification of the Met gene [60]. It would be interesting to assess the potencies and efficacies of Met-targeted PDT on a wider range of Met-expressing tumor cells types. As a consequence of the high expression in MKN45 cells, the receptor is constitutively auto-active and internalized, allowing uptake and intracellular accumulation of the Met-targeted PS [61]. This is in line with what we have observed for G2c-PS and G2c-HL647. As the subcellular localization of PS can influence the mechanism of cell death and the overall potency of the PDT, as suggested in our previous study with different formats of EGFR-targeted VHHs [62], it would be interesting to determine the contribution of PS uptake in the observed effects. The potency and efficacy of the VHH-PS conjugates could be further enhanced by employing a mixture of these Met-targeting conjugates with the previously developed EGFR-targeting ones. This could also affect a larger population of tumor cells, including cells that upregulate Met expression as a resistance mechanism against anti-EGFR therapy, and could be an advantage in tumors with heterogeneously expressed markers such as EGFR and Met. [24,51].

In conclusion, this study has demonstrated that targeting Met using site-directionally conjugated VHH-PS has the ability to specifically kill Met-overexpressing tumor cells. Follow up studies should evaluate the potency of this approach in more relevant models (in vivo). Taken together, Met-targeted PDT might serve as an alternative or complementary approach for combating cancer.
